# Vault Nanocapsules as Adjuvants Favor Cell-Mediated over Antibody-Mediated Immune Responses following Immunization of Mice

**DOI:** 10.1371/journal.pone.0038553

**Published:** 2012-07-11

**Authors:** Upendra K. Kar, Janina Jiang, Cheryl I. Champion, Sahar Salehi, Minu Srivastava, Sherven Sharma, Shahrooz Rabizadeh, Kayvan Niazi, Valerie Kickhoefer, Leonard H. Rome, Kathleen A. Kelly

**Affiliations:** 1 Department of Biological Chemistry, David Geffen School of Medicine, University of California Los Angeles, Los Angeles, California, United States of America; 2 Department of Pathology and Lab Medicine, David Geffen School of Medicine, University of California Los Angeles, Los Angeles, California, United States of America; 3 Molecular Medicine Laboratory, Veteran’s Affairs Greater Los Angeles Healthcare System, Los Angeles, California, United States of America; 4 Department of Bioengineering, Samueli School of Engineering, University of California Los Angeles, Los Angeles, California, United States of America; 5 California NanoSystems Institute, University of California Los Angeles, Los Angeles, California, United States of America; Federal University of São Paulo, Brazil

## Abstract

**Background:**

Modifications of adjuvants that induce cell-mediated over antibody-mediated immunity is desired for development of vaccines. Nanocapsules have been found to be viable adjuvants and are amenable to engineering for desired immune responses. We previously showed that natural nanocapsules called vaults can be genetically engineered to elicit Th1 immunity and protection from a mucosal bacterial infection. The purpose of our study was to characterize immunity produced in response to OVA within vault nanoparticles and compare it to another nanocarrier.

**Methodology and Principal Findings:**

We characterized immunity resulting from immunization with the model antigen, ovalbumin (OVA) encased in vault nanocapsules and liposomes. We measured OVA responsive CD8^+^ and CD4^+^ memory T cell responses, cytokine production and antibody titers *in vitro* and *in vivo*. We found that immunization with OVA contain in vaults induced a greater number of anti-OVA CD8^+^ memory T cells and production of IFNγ plus CD4^+^ memory T cells. Also, modification of the vault body could change the immune response compared to OVA encased in liposomes.

**Conclusions/Significance:**

These experiments show that vault nanocapsules induced strong anti-OVA CD8^+^ and CD4^+^ T cell memory responses and modest antibody production, which markedly differed from the immune response induced by liposomes. We also found that the vault nanocapsule could be modified to change antibody isotypes *in vivo*. Thus it is possible to create a vault nanocapsule vaccine that can result in the unique combination of immunogen-responsive CD8^+^ and CD4^+^ T cell immunity coupled with an IgG1 response for future development of vault nanocapsule-based vaccines against antigens for human pathogens and cancer.

## Introduction

With ongoing disease threats and the promise of emerging immunotherapies, demand for new vaccine technologies is growing. Developing effective and potent vaccines remains one of the most cost-effective strategies for preventing infectious diseases and cancers [1], [2]. Vaccines containing killed or inactivated intact microbes elicit strong immune responses but also produce considerable inflammation at the site of vaccination [3]–[5]. Furthermore, engineered live vaccines, such as non-replicating recombinant viruses have been developed and also induce robust immune responses [6]–[8]. However, the potential for break-through replication of live vectors and anti-vector immunity further discourage the development of live vector vaccines due to safety concerns [9], [10]. To further vaccine development, non-replicating adjuvants are needed which induce robust immunity with minimal inflammation.

The immune-promoting activity of any given vaccination strategy is determined by the presence of the relevant antigenic components in the vaccine formulation, enhanced by the addition of suitable adjuvants capable of activating and promoting an efficient immune response against infectious agents or cancers [1], [2]. One approach for tailoring vaccines to elicit certain types of immune responses while avoiding inflammation is to develop subunit vaccines by combining non-living or synthetic antigens with adjuvants [9]. This type of vaccine can deliver defined antigens with reduced inflammatory cytokine production but is depended on the adjuvant formulation to stimulate cell-mediated immune responses and protection from infectious challenge or prevent tumor growth [11], [12]. Most licensed vaccines promote immunity by eliciting humoral immune responses and weak cellular immune responses. Current efforts are directed to producing adjuvants which elicit cell-mediated immunity [13], [14].

**Figure 1:**
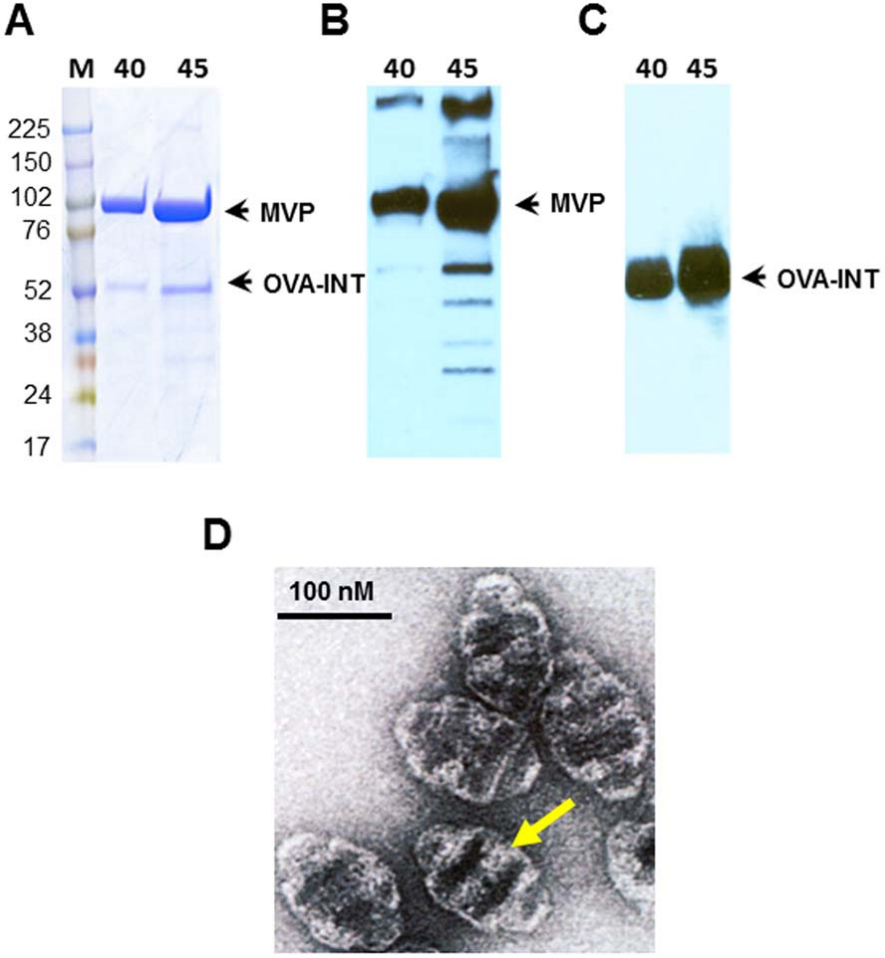
Analysis of purified recombinant vault particles containing OVA-INT. (A) Representative gel image showing co-purification of the protein species MVP and OVA-INT. Sucrose gradients of 40% to 60% run in SDS-PAGE (4%–15%). Lane: M: protein molecular weight markers, 40∶40% fractions of sucrose gradient and 45% fractions of sucrose gradient. (B) The gradient fractions were probed with either anti-MVP rabbit polyclonal antisera or (C) anti-OVA rabbit polyclonal antisera. (D) Negative stain EM of CP-OVA recombinant vaults Bar, 100 nm.

A major limiting factor in the development of subunit vaccines is engineering immune adjuvants to induce cell-mediated immunity and encourage CD8^+^ T cell responses through major histocompatibility complex (MHC) class I presentation (MHC-I, cross presentation). Previous work has shown that it is difficult to achieve antigen presentation through MHC-I molecules unless the antigen is specifically targeted to the MHC-I processing machinery [15]–[17]. A wide range of approaches has been explored including CpG-DNA or toll-like receptor (TLR) ligands, recombinant viral vectors, fusion with bacterial toxins and others [18], [19]. Adjuvants can also be designed to elicit specific immunity, such as promoting cellular immunity which is important for protection against many pathogens [20]. Currently none have been successfully developed for use in humans.

Nanoparticle pharmaceutical carriers can be engineered to elicit various types of immunity and are increasingly investigated as adjuvants for vaccines. Different types of nanocarriers, such as polymers (polymeric nanoparticles, micelles, or dendrimers), lipids (liposomes), viruses (viral nanoparticles), and organometallic compounds (carbon nanotubes) have been employed for immunotherapeutic applications [21]–[23]. We have engineered vaults using a recombinant technique to function as a nanocarrier. Natural vaults are barrel-shaped, hollow, 13 mDa ribonucleoprotein particles that exist in nearly all eukaryotic cells [24], [25]. Their precise function is unknown but they have been associated with multidrug resistance, cell signaling, nuclear-cytoplasmic transport and innate immunity [26]. We have shown that recombinant vaults can be produced to contain a bacterial antigen and induce adaptive immune responses and protective immunity following immunization [27]. In addition, vault nanocapsules can also be engineered to promote anti-tumor responses [28]. These studies show that recombinant vault nanocapsules act as adjuvants, are versatile for eliciting various types of immunity and have outstanding potential for compound encapsulation, protection, and delivery.

This study was performed to characterize the types of immune responses elicited by engineered vault nanopcapsules compared to another type of nanocarrier, liposomes, using a well-characterized model antigen, ovalbumin (OVA). Ovalbumin is a highly immunogenic antigen and has often been used as a proof of principle for numerous vaccination strategies [29], [30]. We show that immunization of mice with OVA encapsulated in vault nanocapsules efficiently stimulates the immune response to elicit robust CD8^+^, CD4^+^ memory T cell responses and antibody titers to OVA. These data support the use of vault nanocapsules as subunit vaccines which can generate both cellular and humoral immunity and provide rationale for using vault nanocapsules to develop vaccines against antigens for human pathogens and cancer.

**Figure 2:**
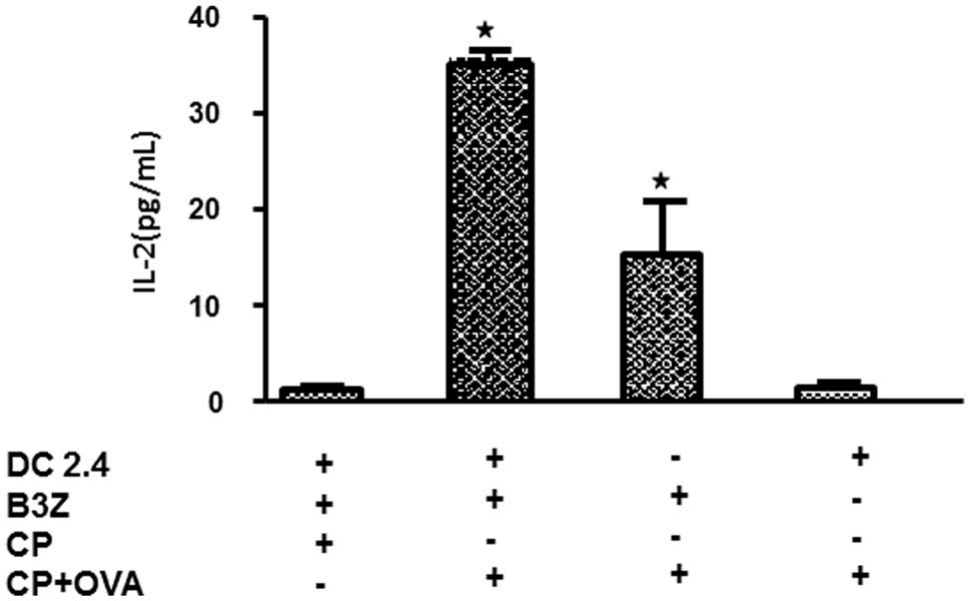
Vault nanocapsules induce cross presentation to CD8 cells. B3Z cells (1×10^5^ cells/200 uL/well) were co cultured with DC 2.4 (5×10^4^ cells/200 µL/well) in the presence or absence of CP-OVA (3.3 µg/200 uL/well) for 24 hrs. Control vaults (CP) were also used at concentration of 3.3 µg/200 uL/well. Following 24 hrs, T cell activation was analyzed by measuring IL-2 production. Data in all panels are representative of 3 independent experiments. Student’s t-test was used to determine statistical significance between the CP-OVA and control CP-vaults. *p<0.05.

## Results

### Preparation of Recombinant Vaults Packaged with Chicken Ovalbumin

Recombinant vaults were produced using a baculovirus expression system in Sf9 insect cells that express a stabilized form of recombinant vaults (CP) and contain a cysteine rich peptide on the N terminus to increase stability [31]. Cryoelectron microscopy imaging of recombinant and tissue derived vaults revealed the localization of the MVP interacting domain, INT [31]. Another form of recombinant vaults (CPZ) contains a 33 amino acid mimic of the Ig binding domain of staphylococcal protein A (Z) in addition to the CP peptide [32]. CPZ vaults were shown to bind antibody and may direct uptake thorough FcRs [27]. These vaults (CP or CPZ) were packaged with chicken ovalbumin by fusion of OVA protein to the vault-targeting protein, INT to form OVA-INT. The OVA-containing vaults were purified and the majority of particles were found in the 40% and 45% sucrose fraction as previously described [33]. Analysis of these fractions by SDS- PAGE and Western blotting (Figure 1) shows the co-purification of MVP and OVA-INT (Figure 1 A). The identity of the components was confirmed by Western analysis with either an anti-MVP polyclonal antibody (Figure 1B) or an anti-OVA antibody (Figure 1C). Purified CP-OVA recombinant vaults were evaluated by negative stain electron microscopy (Figure 1D). The addition of the OVA-INT protein to CP or CPZ did not alter recombinant vault morphology as compared to empty CP vaults when evaluated by transmission electron microcopy (data not shown) and as shown previously [27]. The presence of additional protein density or lighter staining area (arrow) near the waist of the vault barrel, which based on earlier structural studies, is the expected location of OVA-INT [34]. We used these CP and CPZ-vaults containing OVA-INT in vaccinations, henceforth referred to as CP-OVA and CPZ-OVA.

**Figure 3:**
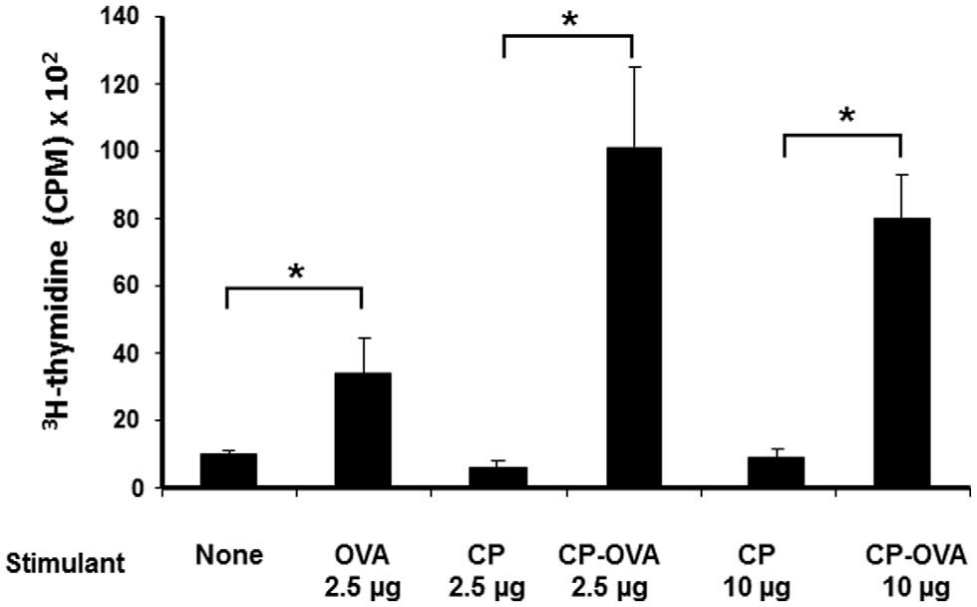
Vault nanocapsules induce CD4 T cell activation. T cells (2×10^5^ cells/mL) were co-cultured with DC (2×10^4^ cells/mL) in the presence of PBS, recombinant OVA protein (2.5 µg/mL), control CP-vaults and CP-OVA with the indicated concentrations. DC-induced T cell proliferation was assessed by incorporation of [^3^H] thymidine. The graphs show mean (SEM) values from a representative experiment (n = 6 replicates) of three independent experiments. Student’s t test was used to determine the p value by comparing appropriate control. *p<0.05.

### Ovalbumin Packaged Inside Vault Nanocapsules can Induce a MHC-I Restricted Response

Dendritic cells (DCs) possess the unique ability to process particulate antigens efficiently into the MHC-I pathway, in a process known as cross-priming. Several approaches have been used to encourage cross priming such as adding exogenous antigenic proteins or peptides with adjuvants to stimulate cytotoxic T lymphocytes (CTLs) [35]. Therefore, we investigated whether recombinant vaults engineered to express OVA could be efficiently internalized, processed and presented by DC in an MHC-I restricted manner to activate CD8^+^ T cells. To this end, the DC2.4 cell line (H-2K^b^) was pulsed with CP-OVA and secretion of IL-2 was measured as an activation marker of the OVA-responsive CD8^+^ T cell hybridoma B3Z (H-2K^b^). The combination of DC2.4 cells, B3Z cells and CP that did not contain OVA-INT could not effectively stimulate IL-2 secretion. However, CP-OVA (produced by combining CP + OVA-INT) incubated with both DC2.4 cells and B3Z hybridoma cells induced secretion of IL-2 (Figure 2). We examined different concentrations of CP-OVA vaults and determined that 3.3 µg CP-OVA vaults per 200 µL per well gave us the greatest IL-2 secretion (data not shown). Additional controls included the B3Z CD8+ T cell hybridoma incubated with CP-OVA alone which induced modest IL-2 levels and suggests that vaults interact with T cells and participate in autopresentation of MHC-I responses [36]. Finally, incubation of CP-OVA vaults with the DC2.4 cell line only produced baseline levels of IL-2. We concluded that exogenous antigen packaged within vault nanocapsules could be delivered and presented by the MHC-I pathway in DCs and possibly through autopresentation to enhance T cell responses.

**Figure 4:**
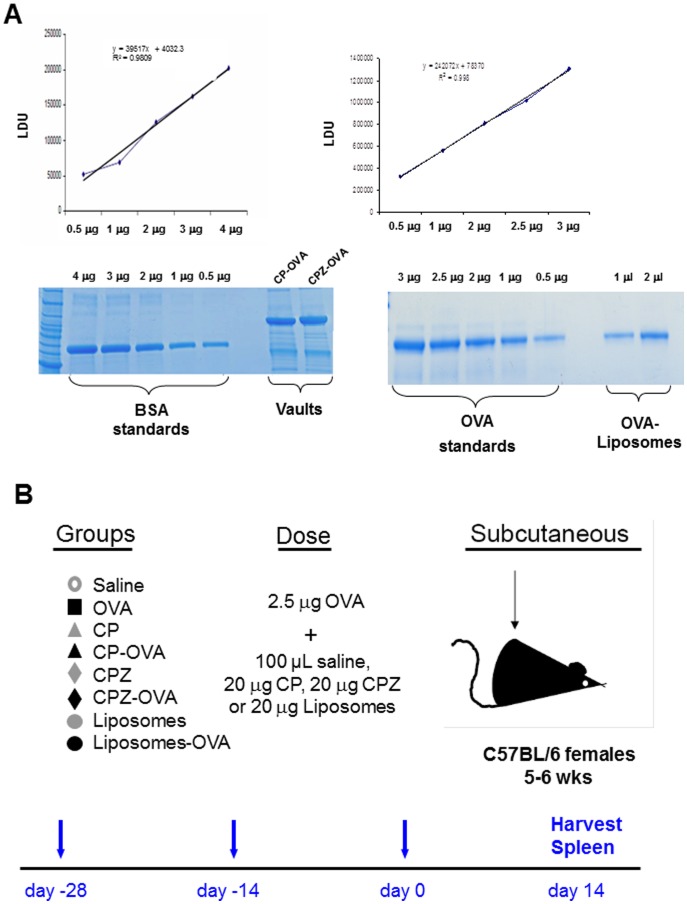
Quantitation of OVA in delivery vehicles and immunization regimen. (A) Images of representative 4–15% SDS polyacrylamide gel showing standards, CP-OVA, CPZ-OVA and OVA-liposomes. The amount of OVA incorporated into the delivery vehicles were quantitated using a Typhoon 9410 Typhoon Variable Mode Scanner of Coomassie blue stained SDS-PAGE gels. (B) Schematic representation of vaccination schedules and subcutaneous immunizations with saline (○), unencapsulated OVA with saline (▪), CP (▴), CP-OVA (▴), CPZ (⧫), CPZ-OVA (⧫), liposome (•), or liposome-OVA (•). The immunization regimen involved three vaccinations (day -28, -14 and 0).

### Ovalbumin Packaged Inside Vault Nanocapsules can Induce a MHC-II Restricted Response

We also examined the MHC class II pathway using bone-marrow–derived DCs from syngeneic BALB/c (H-2 IA/E^d^) mice pulsed with CP-OVA for 24 hours. These DCs were then used to stimulate naive OVA-responsive CD4^+^ T cells from DO11.10 (H-2 A/E^d^) mice. D11.10 cells are transgenic for the TCR recognizing the amino acid 323–339 peptide of OVA on MHC-II. As shown in Figure 3, DC induced significant proliferation in the presence of OVA. However, OVA encased in vault nanoparticles at two concentrations; 2.5 µg and 10.0 µg, stimulated a greater degree of T cell proliferation at both concentrations compared to recombinant OVA protein alone and were not statistically different from each other (Figure 3). These data show that OVA encased in vault nanocapsules was more effective at inducing CD4^+^ T cell proliferation than soluble OVA.

**Figure 5:**
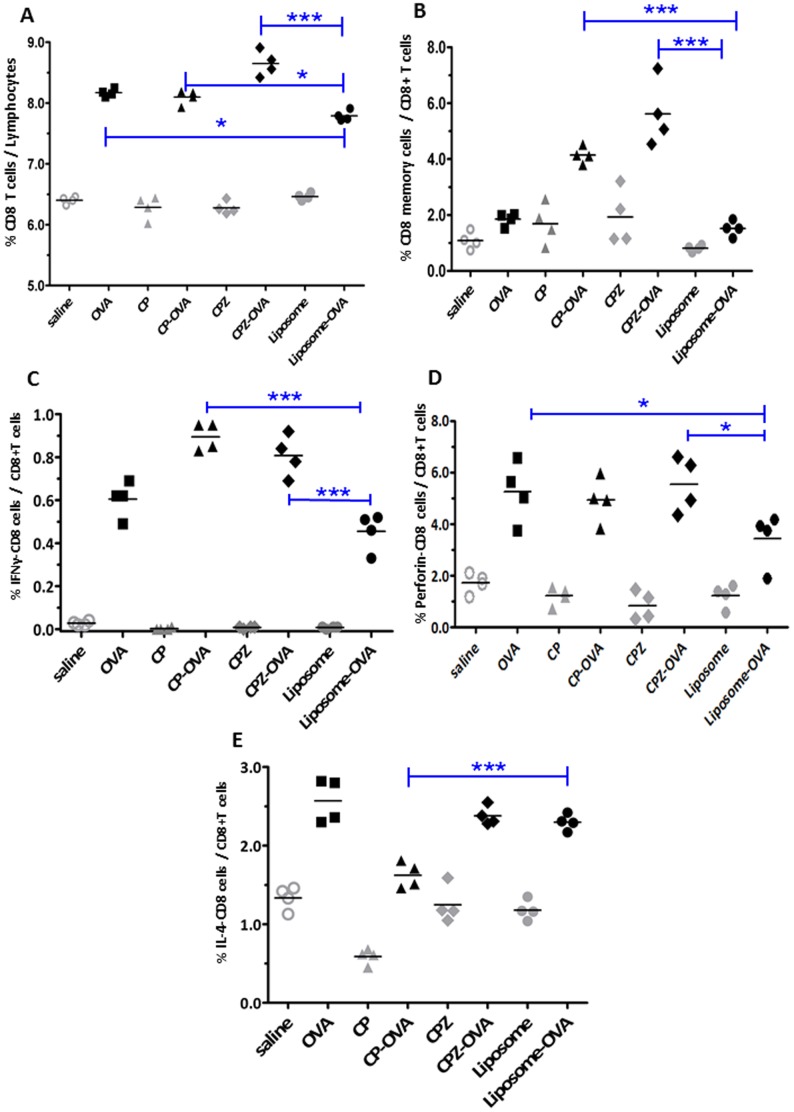
Vault nanocapsules enhance priming of endogenous CD8+ T cells. Mice were injected with various immunogens as shown on the x-axis; saline (○), unencapsulated OVA with saline (▪), CP (▴), CP-OVA (▴), CPZ (⧫), CPZ-OVA (⧫), liposome (•), or liposome-OVA (•). Splenocytes were harvested, stained and gated on lymphocytes as described in the methods section. The frequency of CD8 subpopulations are shown on the y-axis. (A) Total CD8+ cells, (B) CD8+ memory cells (CD8+CD44^hi^), (C) IFNγ-producing CD8+ cells, (D) Perforin-expressing CD8+ cells and (E) IL-4 producing CD8 cells. The cell populations from immunized groups were compared using one-way ANOVA and Bonferroni’s post-hoc test). ***p<0.001, **p<0.01, *p<0.05. Representative of 3 independent experiments.

### Vaccination of Mice with OVA Packaged Vault Nanocapsules Induces CD8^+^ and CD4^+^ T Cells *in vivo*

We characterized cell- and antibody-mediated immune responses to OVA encapsulated in vault nanocapsules and liposomes *in vivo* following subcutaneous administration. To evaluate the type of immune response we immunized mice with either CP-OVA or CPZ-OVA vaults containing equal amounts of endotoxin-free OVA (see material and methods). Liposomes where chosen as a control delivery method since they are a class of nanocarriers and have been utilized as delivery systems for drugs, peptides, proteins and DNA [29], [37]. Liposomes are microscopic vesicles consisting of phospholipid bilayers which surround aqueous compartments and were prepared in this study by encapsulating OVA in DOTAP/DOPE as described in the methods section [38]. The amount of OVA within the vaults and liposomes was quantitated by SDS gel quantitation (Figure 4A). Mice were immunized with equal amounts of delivery vehicle and OVA and the immunization regimen is described in Figure 4B. The percentage of T cells responsive to the OVA CD8 peptide (SIINFEKL) or the OVA CD4 peptide 256–280 (TEWTSSNVMEERKIKV) were documented by surface, intracellular cytokine or perforin staining and FACS analysis after stimulation with each OVA peptide in C57BL/6 mice (H2^b^ background) as described in the methods section. We also examined the anti-OVA-antibody responses following immunization by ELISA.

**Figure 6:**
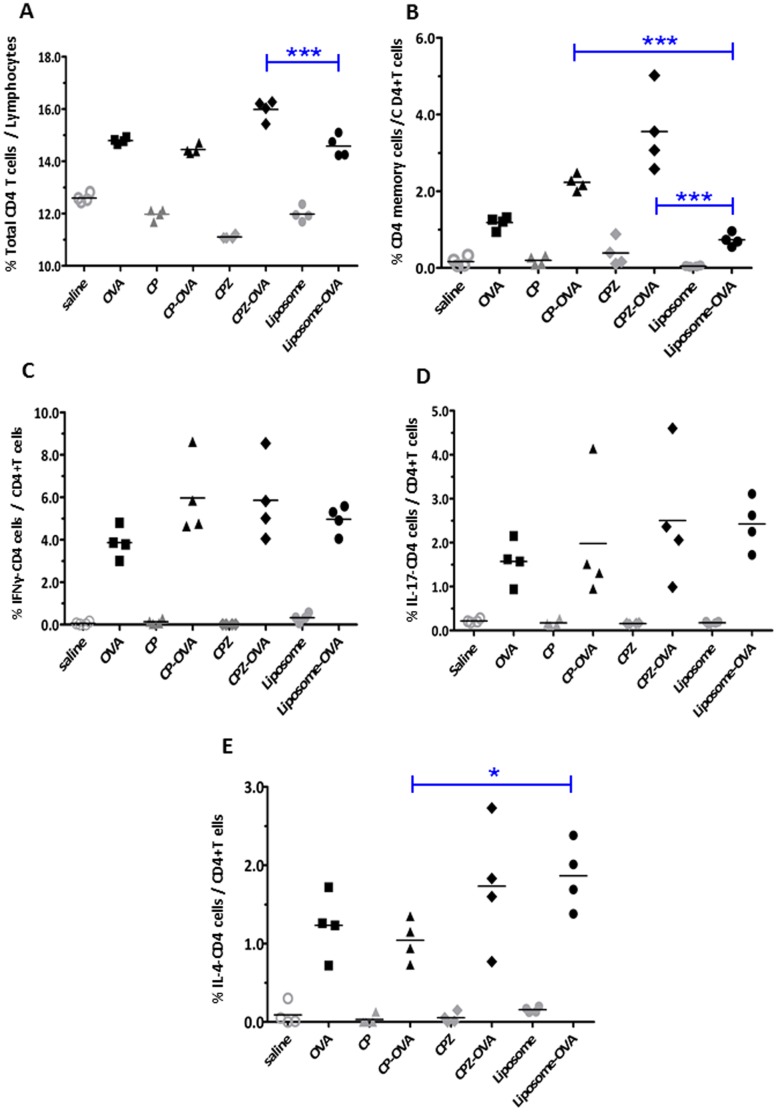
Vault nanocapsules encourage production of CD4+ T cells upon vaccination. Mice were injected with various immunogens as shown on the x-axis; saline (○), unencapsulated OVA with saline (▪), CP (▴), CP-OVA (▴), CPZ (⧫), CPZ-OVA (⧫), liposome (•), or liposome-OVA (•). Splenocytes were harvested, stained and gated on lymphocytes as described in the methods section. The frequency of CD4 subpopulations are shown on the y-axis. (A) Total CD4+ cells, (B) CD4+ memory cells (CD4+CD44^hi^), (C) IFNγ-producing CD4+ cells, (D) IL-17 producing CD4+ cells and (E) IL-4 producing CD4 cells. The cell populations from immunized groups were compared using one-way ANOVA and Bonferroni’s post-hoc test). ***p<0.001, **p<0.01, *p<0.05. Representative of 2 independent experiments.

CD8+ T cells play a critical role in protection against viral and intracellular bacterial and protozoan infections and are important in tumor and graft rejection [39]. After activation, naive antigen (Ag)-responsive CD8^+^ T cells are able to proliferate quickly and differentiate into potent effector cells capable of rapid cytokine production and cytolytic killing of target cells [40], [41]. We wanted to see if entrapment of OVA in vault nanocapsules facilitated cross-presentation of Ag to the MHC-I pathway, resulting in activation of a potent CD8^+^ T cell immunity *in vivo* as we observed previously *in vitro*. We evaluated induction of CD8^+^ T-cell responses among mice immunized with OVA-vaults (CP-OVA and CPZ-OVA), empty vaults (CP and CPZ) and Liposome-OVA as shown in Figure 5. Control groups included soluble OVA and saline immunization. The induction of effector CD8^+^ T cell responses in the spleen was measured 2 weeks after the last immunization by measuring the number of total CD8^+^ T cells, CD8^+^ memory T cells (CD44^hi^), expression of the cytolytic marker perforin, and the production of IFNγ and IL-4 after stimulation with the H2^b^ restricted CD8 OVA peptide, SIINFEKL. All experimental controls were elevated over their respective controls. To simplify the graphs we only show statistical results for comparison of our control immunization group (Liposome-OVA) to the other OVA immunization groups. Our “control” group was Liposome-OVA group because we were interested to learn how vault immunization differed from liposome immunization.

**Figure 7:**
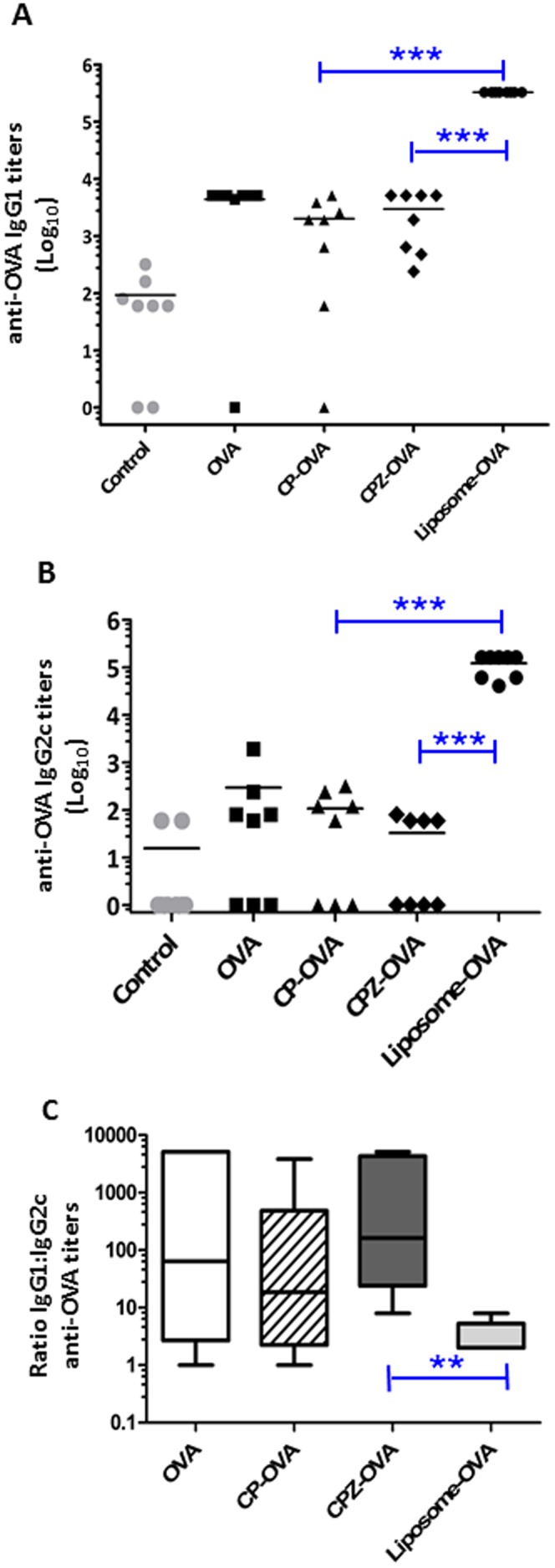
Vault nanocapsules produce lower anti-OVA antibody titers. Antibody titers after vaccination schedule, composed of 3 weekly s.c. injections with control saline (•), unencapsulated OVA in saline (▪), CP-OVA (▴), CPZ-OVA (⧫) or Liposome-OVA (•). (A) Total anti-OVA-IgG1 titers and (B) Total anti-OVA-IgG2c titers. Significance was determined by ANOVA (p<0.001) with Bonferroni post-hoc test (***p<0.001). (C) Ratio of anti-OVA IgG1 to IgG2c antibody. The ratio of Liposome-OVA immunized mice were compared to the other OVA-immunized groups using Mann Whitney t-test (*p<0.001). Data are representative of 2 independent experiments.

As shown in Figure 5A, we found a marked in increase of OVA-responsive SIINFEKL CD8^+^ T cells in the CPZ-OVA immunized group over that found in Liposome-OVA immunized mice in the lymphoid compartment. It was surprising that total CD8^+^ responses were only slightly elevated in the OVA and CP-OVA group and suggested that CD8^+^ T cell subset examination may be more revealing than examining total CD8^+^ T cells in the lymphoid compartment. We also saw an increase in CD8^+^ memory T cells (Figure 5B) and CD8^+^ IFNγ producing T cells (Figure 5C) in mice immunized with OVA encased vault nanocapsules compared to OVA delivered in liposomes while OVA immunization in saline did not increase these responses compared to the Liposome-OVA group. This is consistent with previous studies finding that OVA alone and liposome delivery does not enhance memory CD8^+^ cytotoxic T cells [42]. Although we noted an increase in the number of CD8^+^ T cells expressing perforin in CPZ-OVA immunized mice compared to Liposome-OVA immunized mice we also found increased CD8^+^ perforin^+^ T cells in the OVA group but no increase in the CP-OVA immunized mice. Interestingly, the number of IL-4 producing cells in CP-OVA immunized mice had markedly lower numbers compared to other OVA immunized groups. As expected, vaccination with OVA in any delivery vehicle or dissolved in saline significantly increased SINFEKL-responsive CD8^+^ T cells over control groups for all immunization groups (Figure 5). These findings demonstrate that immunization of antigen encased within vaults is cross-presented *in vivo* and stimulates a CD8^+^ T cell response characterized by memory T cells and IFNγ producing T cells.

It has been documented that CD4^+^ T cell help is important for CD8^+^ T cell function. Since we observed increased numbers of OVA-responsive CD8^+^ memory and IFNγ producing T cells in CP- and CPZ-OVA immunized mice, we investigated if the number of CD4^+^ T cells was also increased following vault immunization. To address this issue, splenocytes from each group were stimulated *ex vivo* with the class II peptide, OVA 265–280 and the CD4^+^ T cell response was characterized by FACS.

We found that immunization with CPZ-OVA but not CP-OVA vault nanocapsules induced a significant amount of total CD4^+^ T cells in the lymphoid compartment of the spleen when compared to Liposome-OVA group (Figure 6A). Also, immunization with both forms of vault nanocapsules significantly elevated the number of CD4^+^ memory T cells compared to Liposome-OVA immunized mice (Figure 6B). We did not see a significant increase in IFNγ or IL-17 producing CD4^+^ T cells over that seen in Liposome-OVA immunized mice following vault or liposome immunization of OVA (Figures 6C & D). However, CPZ-OVA but not CP-OVA immunization induced similar numbers of IL-4 producing CD4+ T cells as mice immunized with Liposome-OVA (Figure 6E). We also noted significant increases in subsets as well as total CD4^+^ T cells in all immunized groups when compared to control groups as expected (Figure 6). Taken together, these data show that immunization with CPZ-OVA induces CD4^+^ T cells characterized by memory cells and IL-4 producing cells. Immunization with CPZ vaults results in the combination CD8^+^ T cells and CD4^+^ helper T cells.

### Vault Nanocapsules can be Modified to Induce Select Antibody Ig Isotypes

Co-operation of CD4^+^ T helper cells with antigen specific B cells is crucial for inducing long-lived neutralizing antibody responses for protective immunity followed by vaccination [43]. We investigated whether OVA delivered in vault nanocapsules also induced anti-OVA antibody since they were capable of inducing CD4^+^ T cell memory and IL-4 producing cells. The serum titers of OVA-responsive IgG1 and IgG2c in each group were measured after immunization by ELISA. We found that mice immunized with Liposome-OVA induced significantly greater levels of anti-OVA IgG1 and IgG2c compared to CP-OVA, CPZ-OVA or OVA immunized mice (Figures 7A & B) indicating that liposomes induce high levels of anti-OVA antibody [44]–[46]. Further inspection revealed that the addition of the “Z” domain reduced mean anti-OVA IgG2c titers by 0.5 to 1 log in comparison to CP-OVA and OVA groups while IgG1 remained comparable. Comparison of the ratio of anti-OVA IgG1:IgG2c revealed that Liposome-OVA immunized mice produced equal levels of IgG1 and IgG2c resulting in a ratio near one while immunization with CP-OVA, CPZ-OVA or OVA increased the ratio of IgG1:IgG2. Moreover, mice immunized with vault nanocapsules modified to express the “Z” domain (CPZ-OVA) had a significantly increased this ratio compared to Liposome-OVA immunized group. In contrast, the OVA and CP-OVA groups were not significantly different compared to the Liposome-OVA group (Figure 7C). As expected all OVA immunization groups induced significant IgG1 and IgG2c serum antibody titers compared to the corresponding controls (Figure 7). These data show that modification of the vault body by addition of the “Z” domain modifies the antibody isotype and suggests that the vault nanocapsule can be modified to alter the humoral responses.

## Discussion

The work presented here illustrates the potential of engineered vault nanocapsules to act as potent adjuvants for the induction of combined cellular and humoral immune responses. Overall, our results demonstrate that immunization of OVA encased in vault nanocapsules, was more effective at generating greater cellular immunity characterized by increased numbers of OVA responsive memory CD8^+^ and CD4^+^ T cells. Also, modification of the vault body, by addition of the “Z” domain, altered the level of anti-OVA Ig subclass as shown by an increased IgG1:IgG2C ratio. These findings show that immune responses against OVA induced by vault nanoparticles differ compared to those induced by liposomes.

An important feature of vault nanocapsules as adjuvants is the robust induction of CD8^+^ and CD4^+^ memory T cells. The delivery of antigens to antigen presenting cells, especially DC, is a critical step for initiating and regulating the adaptive immune responses and we have shown that DC efficiently internalize vault nanocapules [27], [41]. We have also shown that vaults containing immunogenic proteins activate inflammasomes and escape into the cytoplasm [unpublished data, [27]. This may explain induction of an OVA-responsive CD8^+^ memory T cell response and cross-presentation. Vaults may also stimulate antigen-responsive CD8^+^ and CD4^+^ memory T cells by acting as intracellular depots or altering JAK/STAT signaling [47].

A potential vaccine should have the ability to induce and maintain antigen-responsive effector and/or memory T cells [7]. Our data show that immunization with vault nanocapsules was capable of inducing phenotypic markers of memory cells in CD8^+^ and CD4^+^ T cells. It will be interesting to extend these studies and examine memory responses *in vivo* using protection from infection or tumor models. In addition, we found enhanced production of OVA-responsive CD8^+^ T cells that could secrete IFNγ. Surprisingly, there was not much difference between Liposome-OVA and OVA immunized groups and one questions the present of LPS. We did not measure LPS concentrations directly but all reagents used were endotoxin free and the purchased OVA was endotoxin free (see methods). However, there are differences in the amount of IFNγ produced when splenocytes are stimulated with OVA protein, CD8 or CD4 OVA peptides and whether IFNγ is measured in total splenocytes or CD8^+^ or CD4^+^ T cells [48].

The induction of effector CD4^+^ T cells occurs in the same manner and with similar dynamics as is seen with the induction of effector memory CD8^+^ T cells [43]. However, the increased CD4^+^ memory T cells appear to be dominated by helper cells in mice immunized with CPZ-OVA vaults. Our data shows that the addition of the “Z” domain modifies antibody isotypes and supports the increased ratio of anti-OVA IgG1 over IgG2c titers. Adjuvants enhance immunity to immunogens but also steer immunity toward specific immune responses. For instance, alum is a known to promote Th2 responses [49]. The ability of vault vaccines to alter antibody isotypes suggests that modification of the vault toward certain immune responses is possible [50]. Recently, we have modified the vault by the addition of a lytic peptide derived from the adenovirus pIV protein. This modification allows those vaults to rapidly escape phagocytic vesicles [51]. Future studies will examine the *in vivo* immune responses generated by these vaults.

These results plus our previous studies with chemokines (CCL21) [28] and a chlamydial protein (MOMP) [27], supports the hypothesis that vault nanocapsules can be potent antigen delivery vehicles. Vault nanocapsules act as “smart” adjuvants that are capable of directing immunity toward desired responses with little induction of inflammatory cytokines when delivered via a mucosal route [27]. Further studies comparing immunization routes will be needed to determine the most effective route for the desired immune response. Since vaults are ubiquitous and conserved across eukaryote species, the platform has a major advantage over other delivery systems which have safety concerns associated with attenuated bacteria or viruses. In addition, vault nanocapsules are uniform in size and are able to be produced in abundance. Combining adjuvant and carrier activity, engineered vaults enhance the response with a much lower dose of the antigen and circumvent the protein-purification requirements of traditional subunit vaccines and particulate antigen-delivery modalities. With possibilities of further engineering the surface of vaults to either target specific cells or by allowing the proteins to escape endosomes, vaults provide a uniquely tunable platform with ease of manufacture for the delivery of a wide spectrum of subunit antigens for vaccines against infectious disease or other therapeutic targets.

## Materials and Methods

### Ethics Statement

All animal Experimental procedures were approved by the UCLA Institutional Animal Care and Use Committee and conducted according to relevant national and international guidelines. All procedures are designed to provide for maximum comfort/minimal stress to the animals and cannot be further refined to minimize pain/distress since there are no less painful/distressful options available. The procedures are presently refined to provide the best possible scientific methodologies available. The animals are monitored for signs of agitation (licking, biting or guarding the vaginal region), failure to groom, loss of appetite, or marked weight loss (>10%), we will notify the Attending Veterinarian for his/her recommendation for a prophylactic treatment.

### Expression and Purification of Recombinant Vaults

Recombinant baculoviruses were generated using the Bac-to-Bac protocol (Invitrogen, Carlsbad, CA). The 385 amino acid coding region of ovalbumin was fused to major vault protein interaction domain (INT) derived from VPARP (amino acids 1563–1724) by PCR ligation [52], [53]. Two PCR reactions were carried out: OVA-forward :CCCCACTAGTCCATGGGCTCCATCGG and OVA-INT reverse: TCCTGCCAGTGTTGTGTGCAGCTAGCAGGGGAAACACATCTGCC using plasmid pMFG-OVA as the template (plasmid pMFG-OVA was a kind gift from Dr Carlo Heirman, Laboratory of Molecular and Cellular Therapy, Department of Physiology–Immunology, Medical School of the Vrije Universiteit Brussel, Brussels, Belgium). The second PCR reaction with primer OVA-INT forward: TTGGCAGATGTGTTTCCCCTGCTAGCTGC ACACAACACTGGCAGGA and INT reverse: GGGCTCGAGTTAGCCTTGACTGTAATGGAG using INT in pET28 as the template. The PCR reactions were purified on a Qiagen column and a second round of PCR was carried out using the OVA-forward × INT reverse. The resultant PCR product containing the fused OVA-INT was purified on a Qiagen column, digested with Spe I and Xho I, gel purified, and ligated to pFastBac to form a pFastBac vector containing OVA-INT. Construction of cp-MVP-z, or cp-MVP in pFastBac has been described previously [32].

Sf9 cells were infected with Ova-INT, cp-MVP-z, or cp-MVP recombinant baculoviruses at a multiplicity of infection (MOI) of 0.01 for approximately 65 h and then pelleted and lysed on ice in buffer A [50 mM Tris-HCl (pH 7.4), 75 mM NaCl, and 0.5 mM MgCl2] with 1% Triton X-100, 1 mM dithiothreitol, 0.5 mM `µg/ml chymostatin, 5 µM leupeptin, 5 µM pepstatin) (Sigma, St. Louis, MO). Lysates containing cp-MVP-z vaults were mixed with lysates containing either OVA-INT were incubated on ice for 30 min to allow the INT fusion proteins to package inside of vaults. Recombinant vaults were purified as previously described [33] and resuspended in 100–200 µl of sterile phosphate buffered saline. The protein concentration was determined using the BCA assay (Pierce, Rockville, IL) and sample integrity was analyzed by negative stain electron microscopy and SDS-PAGE with Coomassie staining or transferred to hybond membrane (Amersham) for Western blot analysis. The density of the bands was determined by gel scanning and densitometry analysis using a 9410 Typhoon Variable Mode Scanner (GE Healthcare Life Sciences, Piscataway, NJ).

### Preparation of OVA-liposomes

To generate OVA-liposomes, 10 mg lyophilized DOTAP/DOPE (1∶1) (1,2-dioleoyl-3-trimethylammonium-propane/1,2-dioleoyl-*sn*-glycero-3-phospho-ethanolamine) (Avanti Polar Lipids, Alabaster, AL) was re-hydrated in 1 mL endotoxin-free 5% glucose and mixed slowly (rotated) overnight at room temperature. Lyophilized EndoGrade Ovalbumin (<1 EU/mg i.e. 1 endotoxin unit has ∼0.1 ng of endotoxin) (Profos AG, BioVender, LLC, Candler, NC) was reconstituted in endotoxin-free sterile saline (<0.1 EU/mL endotoxin, Sigma) to a stock solution of 10 mg/mL. Aliquots were stored frozen and thawed immediately before use. The entrapment of OVA was generated by combining 1.25 mg of resuspended ovalbumin with 2.5 mg of swollen DOTAP/DOPE lipids and further facilitated by brief sonication. OVA-liposomes were separated from unincorporated ovalbumin by ultracentrifugation at 100,000×g using an Optima XL-80K (Beckman Coulter, Fullerton, CA) ultracentrifuge and washed two additional times. Quantitation of encapsulated OVA was determined by subjecting OVA-liposomes (1, 2, 4 µL) to SDS-PAGE electrophoresis in parallel with known amounts of ovalbumin (0.25, 0.5, 1.0, 2.5, 5 µg) and visualized by Coomassie blue staining.

### Gel Electrophoresis and Immunoblotting

Sodium dodecyl sulfate-polyacrylamide gel electrophoresis was performed using the discontinuous buffer system and 4–15% acrylamide gels. Protein samples of OVA-liposome or OVA-vaults were transferred to an Immobilon-P transfer membrane (Millipore, city, Bedford, MA) and blocked with 5% nonfat dry milk in PBS-0.1% Tween 20 (PBS-T). Membranes were incubated for 1 hr with anti-MVP (1∶500, MAB 1023, Santa Cruz Biotechnology Inc, Santa Cruz, CA) or anti-INT followed by a 1 h incubation with the appropriate horseradish conjugate (1∶5,000, Amersham Biosciences, Piscataway, NJ). Bound conjugates were detected with ECL-Plus (GE Healthcare, Life Sciences, Piscataway, NJ) and 9410 Typhoon Variable Mode Scanner (GE Healthcare Life Sciences, Piscataway, NJ).

### Antigen Processing and Presentation Assay

DC2.4 H-2Kb (5×10^4^/well) were plated in triplicates in 96-well plates and allowed to settle at 37°C. Then, MHC Class I restricted CD8^+^ T cell line B3Z (10^5^/well) were added, in the presence of control vaults (200 ng/mL) and OVA vaults (200 ng/mL) for 24 hrs. After 24 h incubation at 37°C, the plate was centrifuged at 1800 rpm, and the culture supernatant was collected and assayed for IL-2 using an IL-2 ELISA kit (BD Biosciences, San Jose, CA).

### DC-dependent T Cell Proliferation

DC cultures were generated by flushing the bone marrow (BM) from the bone shafts, washed and plated bacteriological Petri dishes (Falcon Plastics, Oxnard, CA). The cells were cultured at 2×10^5^ cells/mL in RPMI 1640 culture medium (10 mM HEPES/2 mM l-glutamine/10% 0.22 um filtered FBS/50 uM β-mercaptoethanol) supplemented with mGM-CSF (20 ng/mL) and mIL-4 (20 ng/mL) in an atmosphere of 5% CO_2_ at 37°C. Fresh medium containing mGM-CSF (20 ng/mL) and mIL-4 (20 ng/mL) was added for 3–6 days after the start of culture. To induce maturation, cells were cultured for an additional 24 h in the presence of LPS (1 µg/mL). The DC were harvested and purified with anti-CD11c magnetic beads, and suspended in complete RPMI-1640 medium and seeded at 5×10^5^/mL/well on 24-well culture plates followed by incubation with 25 and 100 µg/mL of CP-OVA or recombinant OVA protein for 4 h at 37°C, 5% CO_2_. Nonadherent cells consisting of mostly immature or mature DC were harvested for all the analyses performed in this study. Responder CD4^+^ T cells were separated from splenocytes with mouse CD4^+^ T-cell enrichment system (StemCell Technologies, Vancouver, Canada) according to the manufacturer’s instructions. CD4^+^ T cells (2×10^4^/well) were added to OVA protein or CP-OVA pulsed DC and cultured for an additional 4 days. During the last 16–18 h of the 4-day culture, cells were pulsed with 1 µCi [3H]thymidine (Amersham, Arlington, IL). The cells were harvested onto filter paper and [3H]thymidine incorporation was measured with a β-plate scintillation counter (PerkinElmer, Wellesley, MA).

### Immunization Procedures

The OVA protein concentration was adjusted using endotoxin-free sterile saline (<0.1 EU/mL, 1 EU has ∼0.1 of endotoxin (Sigma) to 2.5 µg OVA in 20 µg of vault nanoparticles or liposomes using a Typhoon 9410 Variable Mode Scanner of Coomassie blue stained SDS-PAGE gels. The immunogens were injected into C57BL/6 mice (5–6 wk old) by subcutaneous injections at the base of the neck in 100 µl sterile saline. The mice were immunized 3 times at 2 wk intervals. The spleen and blood was obtained 2 wk after the last immunization. The splenocytes were immediately used for FACS analysis and serum samples were stored frozen at −80°C until assayed.

### Measurement of anti-OVA Antibody from Serum

An ELISA was used to determine the level of anti-OVA antibody isotypes in the serum. Briefly 96-well microtitre plates (Nunc, Roskilde, Denmark) were coated with 75 µl per well of OVA (1 µg/75 µl) in PBS and incubated over night at 4°C. After being washed in buffer (phosphate buffered saline containing 0.05% Tween-20 (v/v) (PBS/T20) the plates were blocked with 150 µl of PBS supplemented with 5% non-fat dry milk for 2 h at room temperature. After washing, 7 µl of serum diluted from 1∶40 to 1∶5120 in PBS was added and incubated at 4°C overnight. Unbound antibody was then washed away and 75 µl of goat anti-mouse IgG1- IgG2c-biotin (Southern Biotechnology Associates, Inc., Birmingham, AL), diluted 1/10,000 in PBS, was added and the plates incubated for 4 h at room temperature. The plates were then washed and 75 µl of NeutraAvidin horse radish peroxidase diluted in PBS at 1∶1000 was added for 20 min. After a final wash step, 100 mL of TetraMethylBenzidine (TMB) (Zymed Laboratories Inc., San Francisco, CA) substrate was added and incubated at room temperature, in the dark, for 20 min. The reaction was stopped with 50 µL of 2 N sulphuric acid and the plates were read at 450 nm in a microplate reader (Model 550, Bio-Rad Laboratories, Hercules, CA).

### Measurement of IL-2 Production

Spleens were removed and placed in RPMI media (Gibco, Grand Island, NY) supplemented with 10% heat inactivated FCS. They were macerated to release the lymphocytes which were then washed by centrifugation. The cell pellet was resuspended in fresh media at a concentration of 2×10^6^ cells/mL and 1 mL of cells placed in each well of a 24-well plate (Nunc, Roskilde, Demark). They were restimulated with media (negative control) or OVA (100 µg/mL) for 72 h at 37°C in a humidified atmosphere with 5% CO_2_. The plate was frozen until required. One hundred microlitres of the supernatants were tested for IL-2 in a sandwich ELISA following the manufacturer’s instructions (PharMingen, San Diego, USA). In brief, 96-well, flat-bottomed plates were coated with 50 µL of a 2 µg/mL concentration of capture antibody (PharMingen). Plates were washed and blocked with 200 µL/well of PBS/FCS. Doubling dilutions of standards and supernatants were added and incubated at 4°C overnight. The plates were washed and 100 µL of a biotin-conjugated detecting mAb (PharMingen) was added at a concentration of 1 mg/mL. The enzyme and substrate were then added and analyzed as per the serum antibody ELISA. The amount of each cytokine in the supernatant was extrapolated from the standard curve derived using recombinant IL-2 (PharMingen) standards.

### Characterization of T Cell Populations by Flow Cytometry

Lymphocytes were isolated from spleens by mechanical disruption through a cell strainer. RBCs were lysed using ammonium chloride-potassium buffer. The cells were stimulated at 37°C with OVA peptide 265–280:TEWTSSNVMEERKIKV (2 µg) to identify CD4 cells or OVA peptide: SIINFEKL (2 µg) to identify CD8 cells for 5 hr. For the last 4 h, cells were incubated in the presence of Brefeldin A (BioLegend) at 1 µg/mL. At the end of culture, the cells were stained using fluorochrome-conjugated MAbs against CD3, CD8, CD4, CD44, CCR7 and CD62L (BioLegend, San Diego, CA) in staining buffer (PBS with 2% fetal bovine serum and 0.1% sodium azide) and then treated with Fix/Perm (BioLegend). After permeabilization, the cells were further stained with fluorochrome-conjugated antibodies against IFN-γ, IL-4, IL-17 and perforin. Data were collected on LSR II (BD Biosciences, San Jose, CA) and analyzed using FCS Express (De Novo Software, Los Angeles, CA). CD8^+^ and CD4^+^ T cells were determined by gating on lymphocytes (FSC vs SSC) and CD8^+^ or CD4^+^ memory, cytokine producing or perforin expressing T cells were determined by gating on either CD3^+^CD8^+^ or CD3^+^CD4^+^ T cells as shown in Figure S1.

### Statistical Analysis

Statistical analysis was performed using Prism 5 (GraphPad, San Diego, CA). Data are presented as mean for each group and statistical significance for IL-2 secretion, proliferation, flow cytometry and Ig titers were determined by one way analysis of variance (ANOVA) with Bonferroni’s Multiple Comparison Test. The ratio of isotypes was compared by Kruskal-Walis and Dunn’s post-test.

## Supporting Information

Figure S1 **Flow cytometry gating scheme used to define cell populations.** (A) A representative dotplot from a CPZ-OVA immunized mouse was gated on lymphocytes using SSC versus FSC. The percent of CD3^+^CD8^+^ memory T cells was determined from the events in the lymphocyte gate. (B) The lymphocyte gated population was further gated on CD3^+^ T cells and CD3^+^ T cells were separated into CD8^+^ or CD4^+^ T cells. Memory cell population was determined by hi expression of CD44 and a gate drawn. This was applied to all experimental mice to determine the percentage of CD8^+^ memory cells. The scheme was applied to CD8^+^ or CD4^+^ T cells producing cytokines or expressing perforin by gating on the CD3^+^CD8^+^ or CD3^+^CD4^+^ population.
(TIF)
