# Supplementary figures and images for: Vault Nanocapsules as Adjuvants Favor Cell-Mediated over Antibody-Mediated Immune Responses following Immunization of Mice
Source: PLoS One. 2012 Jul 11;7(7):e38553. doi: 10.1371/journal.pone.0038553 (PMC3394761; doi:10.1371/journal.pone.0038553)

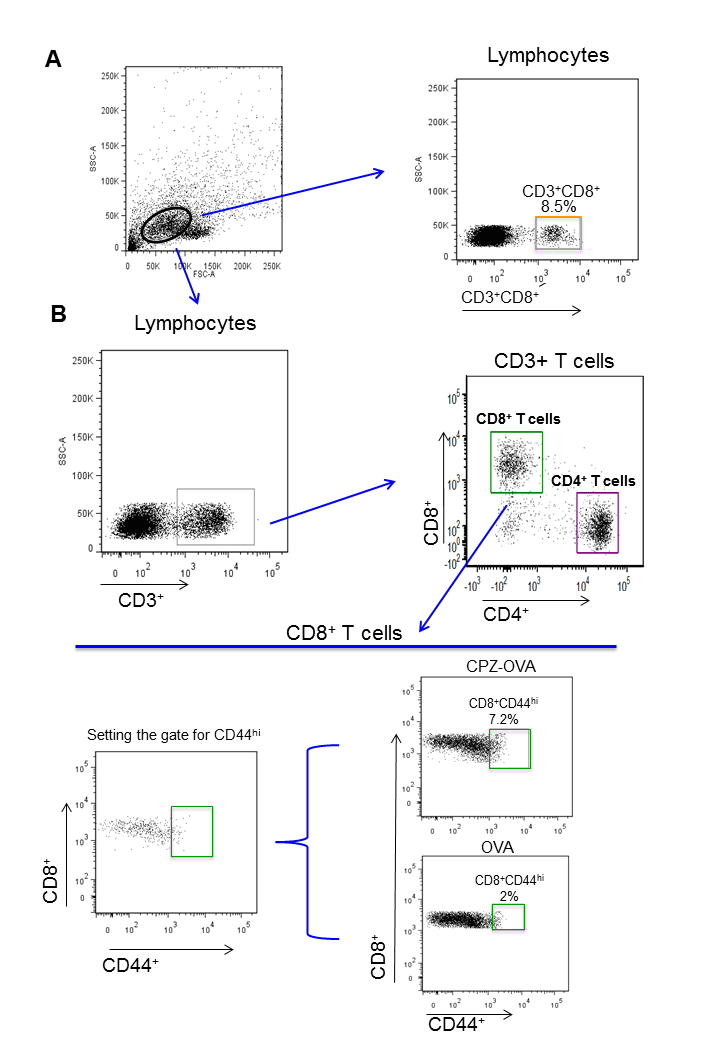

Supplement: Figure S1 — Flow cytometry gating scheme used to define cell populations. (A) A representative dotplot from a CPZ-OVA immunized mouse was gated on lymphocytes using SSC versus FSC. The percent of CD3+CD8+ memory T cells was determined from the events in the lymphocyte gate. (B) The lymphocyte gated population was further gated on CD3+ T cells and CD3+ T cells were separated into CD8+ or CD4+ T cells. Memory cell population was determined by hi expression of CD44 and a gate drawn. This was applied to all experimental mice to determine the percentage of CD8+ memory cells. The scheme was applied to CD8+ or CD4+ T cells producing cytokines or expressing perforin by gating on the CD3+CD8+ or CD3+CD4+ population. (TIF) [file pone.0038553.s001.tif]
